# Native Cadmium-Tolerant *Trichoderma* Associated with the Rhizosphere of *Inga* sp. and *Quararibea* sp. in Amazonian Cocoa Agroecosystems of Peru

**DOI:** 10.3390/jof12090644

**Published:** 2026-09-01

**Authors:** Jessenia Shirley Ramos Armaulia, Lizette Daniana Méndez Fasabi, Santos Triunfo Leiva Espinoza, Segundo Manuel Oliva Cruz

**Affiliations:** 1 Instituto de Investigación Para el Desarrollo Sustentable de Ceja de Selva (INDES-CES), Universidad Nacional Toribio Rodríguez de Mendoza, Chachapoyas 01001, Amazonas, Peru; santos.leiva@untrm.edu.pe; 2 Escuela de Ingeniería Agronómica, Facultad de Ingeniería y Ciencias Agrarias, Universidad Nacional Toribio Rodríguez de Mendoza, Chachapoyas 01001, Amazonas, Peru; lizette.mendez@untrm.edu.pe

**Keywords:** heavy-metal tolerance, dose–response modeling, IC_50_, multilocus phylogeny, growth inhibition percentage, fungal bioremediation, shade-tree rhizosphere, *Theobroma cacao*

## Abstract

Cadmium (Cd) contamination of Amazonian cocoa soils threatens *Theobroma cacao* production, and shade-tree rhizospheres may harbour Cd-tolerant fungi useful for bioremediation. *Trichoderma* associated with *Quararibea* sp. remain unexplored. We isolated native *Trichoderma* from rhizospheres of *Inga* sp. and *Quararibea* sp. in Bagua (Peru), and evaluated Cd tolerance (25–200 ppm) through growth inhibition percentage (GIP) and IC_50_ from dose–response models selected via the Akaike Information Criterion (AIC). Of 16 *Trichoderma*-like isolates, five met stringent multilocus quality criteria: JSRA09 (*Quararibea* sp.) and JSRA16, JSRA22, JSRA28, JSRA30 (*Inga* sp.). Independent Maximum Likelihood trees (ITS, *tef1*, *rpb2*) assigned JSRA09 to the *T. spirale*/*T. subuliforme* clade, JSRA16 to the *T. harzianum* complex (affinity to *T. lentiforme*), JSRA22 and JSRA28 to a clade within the *T. koningii*/*T. koningiopsis* complex, and JSRA30 to *T. afroharzianum*. To the best of our knowledge, JSRA09 represents the first *Trichoderma* report in *Quararibea* sp. No strain exceeded 35% inhibition at 200 ppm: JSRA09 and JSRA16 were highly tolerant (GIP < 10%), JSRA28 and JSRA30 moderately tolerant, and JSRA22 tolerant. AIC selection yielded linear responses for JSRA09 and JSRA16, log-logistic (LL.4) for JSRA22, JSRA28 and JSRA30. These native strains are candidates for further screening towards microbial consortia for Cd bioremediation.

## 1. Introduction

Cadmium (Cd) contamination of Amazonian cocoa soils represents a direct threat to the sustainability of *Theobroma cacao* production in the province of Bagua, Amazonas, Peru. Local studies report Cd concentrations in soils ranging from 1.02 to 3.54 mg kg^−1^, with an average of 1.7 mg kg^−1^ [1], values that exceed the Peruvian Environmental Quality Standard for agricultural soils (1.4 mg kg^−1^) [2]. This edaphic contamination leads to grains with Cd contents that exceed the limits set by Regulation (EU) 2023/915 [3] for cocoa-derived products, compromising commercialization under the “Cacao Amazonas Perú” Denomination of Origin and posing a public health risk through the entry of the metal into the food chain [1]. Cd is efficiently absorbed by cacao roots and translocated to aerial tissues, with variable concentrations in roots, leaves and cotyledons that depend on an acidic soil pH and on local geochemical factors.

In the cocoa agroforestry systems of Bagua, shade trees play a key structural and ecological role. A typical plot usually contains around six *Inga* sp. (Fabaceae) trees and two *Quararibea* sp. (Malvaceae) trees, although composition varies between plots and districts: in some sites both species coexist, while in others only *Inga* sp. is found, generating heterogeneous rhizosphere microenvironments at the local scale. *Inga* sp., a leguminous shade tree commonly used in cocoa agroforestry, develops a fine-root system that overlaps spatially with cacao roots in the upper soil layers [4] and harbours a rich rhizosphere mycobiome, including arbuscular mycorrhizal fungi, in Amazonian agroecosystems [5]; however, few studies have examined its potential as a reservoir of heavy-metal-tolerant microorganisms in Amazonian contexts. *Quararibea* sp., in turn, has been studied mainly for the accumulation of minerals (Fe, Na, Mg, Cu) in its fruits [6], but its rhizosphere remains unexplored regarding fungal communities adapted to Cd stress.

The rhizosphere of plants growing in heavy-metal-contaminated soils acts as a selective ecological niche that promotes the proliferation of tolerant microorganisms capable of modulating metal bioavailability through immobilization, bioaccumulation and stimulation of plant growth [7,8]. The genus *Trichoderma* stands out in this context for its metabolic versatility, its capacity to produce chelating compounds and its potential to mitigate heavy-metal phytotoxicity, reducing Cd and Pb mobility in the rhizosphere and improving plant growth under metal stress [9,10]. However, most studies have been conducted under controlled conditions or in non-Amazonian soils with non-native strains, which limits the direct transfer of results to Peruvian cocoa agroecosystems. Although three endemic *Trichoderma* species have been described from cocoa soils in Amazonas (*T. awajun*, *T. peruvianum* and *T. jaklitschii*) [11], their differential Cd tolerance has not been evaluated.

In this context, no previous reports exist on the isolation and molecular identification of *Trichoderma* in the rhizosphere of *Quararibea* sp., which constitutes a specific knowledge gap for Amazonian cocoa agroecosystems. The heterogeneity in tree composition between plots and districts (Imaza and Copallín, for example) suggests that the rhizospheres of *Inga* sp. and *Quararibea* sp. may harbour fungal lineages with local adaptations to acidic edaphic conditions and Cd presence. The present study had the following objectives: (i) to morphologically and molecularly characterize, through a multilocus phylogenetic approach (ITS, *tef1*, *rpb2*), the native *Trichoderma* strains isolated from the rhizosphere of *Inga* sp. and *Quararibea* sp. in cocoa plots of Bagua; (ii) to evaluate their Cd tolerance (25–200 ppm) through radial growth parameters, growth inhibition percentage (GIP), tolerance index (TI) and median inhibitory concentration (IC_50_) estimated from dose–response models selected via the Akaike Information Criterion (AIC) [12]. This in vitro study is conceived as a primary screening stage whose results should guide future evaluations under soil and field conditions. We hypothesized that these rhizospheres harbour strains with differential Cd tolerance, possibly including lineages with affinity to those previously described in the region, positioning them as candidates for further screening in Cd-contaminated soils and for the eventual development of native microbial consortia in bioremediation strategies adapted to Amazonian cocoa agroecosystems.

## 2. Materials and Methods

### 2.1. Study Area and Soil Cadmium Levels

**Figure 1:**
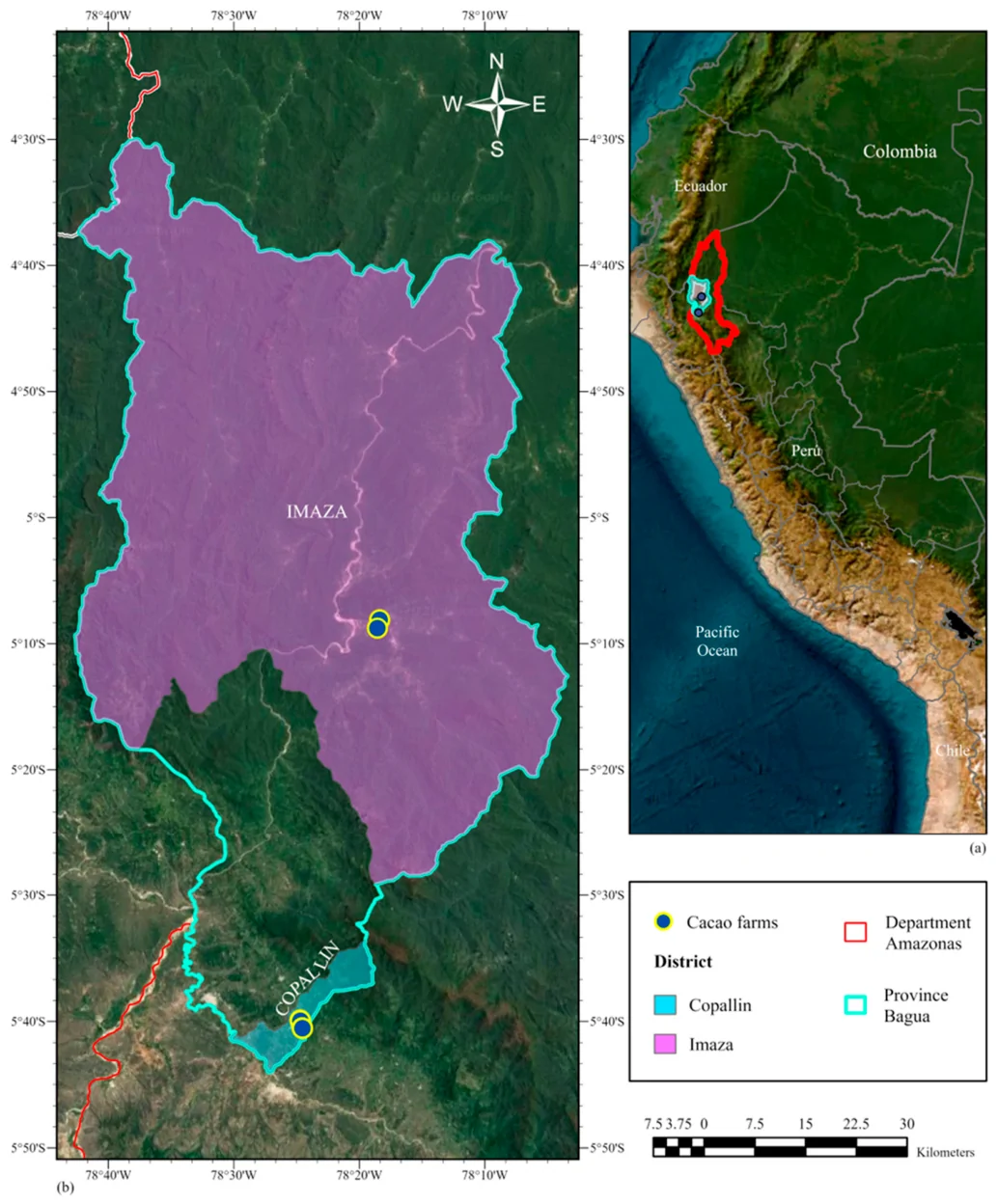
Location map of the study area in Bagua, Amazonas, Peru: (**a**) location of the Amazonas Department and Bagua Province within Peru; (**b**) study area showing the cocoa agroforestry plots in the districts of Imaza and Copallín (verified by ArcGIS Pro georeferencing) where rhizosphere soil samples were collected from *Inga* sp. and *Quararibea* sp. trees.

This study was conducted in cocoa (*Theobroma cacao*) plots under agroforestry systems in Bagua, Amazonas, Peru (Figure 1). Plots were selected based on two criteria: (i) historical Cd contamination of the soil and (ii) the presence of *Inga* sp. and *Quararibea* sp. as shade trees. The sampling area is distributed across two administrative districts of the Bagua Province (Imaza and Copallín), as verified by ArcGIS Pro v3.4.0 (Esri, Redlands, CA, USA) georeferencing of GPS coordinates recorded during field sampling. Cd levels in these plots ranged from 1.02 to 3.54 mg kg^−1^ at 0–30 cm depth (mean: 1.7 mg kg^−1^) [1], values that exceed the Peruvian Environmental Quality Standard for agricultural soils (1.4 mg kg^−1^) [2].

### 2.2. Plant Species and Rhizosphere Sampling

Rhizosphere soil sampling was performed following the protocol established in the Peruvian Soil Sampling Guide [13]. Using a hand auger and sterile spade, samples were collected at a depth of 10–20 cm at four cardinal points around each selected tree (*Inga* sp. and *Quararibea* sp.), at a distance of 50 cm from the main trunk. At each point, approximately 100 g of soil was collected; the four sub-samples were homogenized to obtain a composite sample of 400 g per individual tree. Samples were stored in sterile labelled bags and transported under refrigerated conditions (4–10 °C) for subsequent analysis.

A total of 36 rhizosphere soil samples were collected across the studied agroforestry plots. In the typical agroforestry composition of these plots, *Inga* sp. is the dominant shade tree species (approximately six individuals per plot), whereas *Quararibea* sp. is present at a lower density (approximately two individuals per plot, and absent in some plots). The 36 samples were distributed across host species as follows: 31 samples from *Inga* sp., 3 samples from *Quararibea* sp., and 2 additional samples from other shade tree species (one *Erythrina* sp. and one unidentified tree); the latter two were excluded from molecular analyses because they did not fit the focal host pair of this study. This sampling distribution reflects the natural composition of the agroforestry plots rather than an experimental design choice, and is acknowledged as a limitation in Section 4.5. GPS coordinates were recorded at each sampling point with a handheld GPS receiver in UTM 17M coordinates, and the corresponding administrative districts (Imaza and Copallín, Bagua Province) were verified by ArcGIS Pro georeferencing during data processing. Sampling coordinates, altitudes and host plant assignments for the 16 *Trichoderma*-like isolates that were ultimately recovered are detailed in Supplementary Table S4.

### 2.3. Isolation and Morphological Characterization of Trichoderma

Isolation of *Trichoderma* spp. from rhizosphere soil samples was performed using Potato Dextrose Agar (PDA) medium supplemented with antibiotics (rifampicin, streptomycin and chloramphenicol), following Elad et al. [14] and Samuels [15]. Ten grams of each soil sample were suspended in 90 mL of sterile distilled water. The suspension was vigorously shaken for 30 min and serial dilutions (10^−1^ to 10^−4^) were prepared. Aliquots of 1 mL from the 10^−3^ and 10^−4^ dilutions were inoculated on Petri plates with selective medium and incubated at 25–28 °C for 5–7 days. Colonies with typical *Trichoderma* spp. characteristics (rapid growth and green pigmentation) were sub-cultured on pure PDA medium. Of the 36 rhizosphere samples processed, 16 yielded *Trichoderma*-like isolates on the selective medium (Supplementary Table S4); the remaining 20 samples produced colonies of other soil fungi (*Fusarium*, *Mucor*, *Alternaria*, *Aspergillus*, among others) without typical *Trichoderma* morphology, and were not processed further in this study. The 16 *Trichoderma*-like isolates were subjected to subsequent molecular identification (Section 2.4). Macroscopic features (colour, texture, growth pattern) and microscopic features (morphology of hyphae, conidiophores and conidia) were recorded for the isolates that passed multilocus identification criteria. For microscopic observation, temporary mounts were prepared with lactophenol cotton blue and examined at 10×, 40× and 100× following the taxonomic keys of Gams and Bissett [16] and Jaklitsch [17].

### 2.4. Molecular Identification Through a Multilocus Approach (ITS, tef1, rpb2) and Sequence Quality Control

DNA extraction was performed from pure *Trichoderma* cultures incubated for 48–72 h on PDA at 28 °C. Mycelium was collected and DNA was extracted using the NucleoSpin Microbial DNA kit (Macherey-Nagel, Düren, Germany), following the manufacturer’s instructions. DNA concentration and purity were determined by spectrophotometry (Eppendorf BioSpectrometer kinetic, Hamburg, Germany). PCR amplification was performed for the ITS, *tef1* and *rpb2* markers using the primers and conditions described by Cai and Druzhinina [12] and Samuels [15]. PCR products were verified by electrophoresis on 1% agarose gel stained with SYBR Safe, and the amplified bands were sent for commercial sequencing to Macrogen (Santiago, Chile).

All 16 isolates were subjected to PCR amplification and Sanger sequencing of the three phylogenetic markers. Isolates were retained for downstream phylogenetic and Cd tolerance analyses only when two conditions were simultaneously met: (i) high-quality bidirectional Sanger reads were obtained for all three markers (ITS, *tef1*, *rpb2*); (ii) the multilocus BLAST (https://blast.ncbi.nlm.nih.gov, accessed on 20 August 2025) assignments were congruent across markers at the species- or clade-level, in accordance with the multilocus identification criteria of Cai and Druzhinina [12]. Specifically, the protocol requires that the *rpb2* best BLAST hit (≥99% similarity) and the *tef1* best BLAST hit (≥97% similarity) converge on a single species or, when this is not achievable due to known cryptic complexes within *Trichoderma*, on a well-defined species complex with consistent membership across markers. Of the 16 isolates, eleven failed the retention criteria for one of two reasons: eight isolates (JSRA18, JSRA25, JSRA27, JSRA31, JSRA32, JSRA34, JSRA35 and JSRA25x) showed sequencing quality below the Q ≥ 50% threshold in Sequencher v5.4.6 for one or more markers (most commonly *tef1* or ITS, which are known to produce variable amplification quality in *Trichoderma* [12]); three isolates (JSRA15, JSRA20 and JSRA09C1) produced high-quality sequences in all three markers but showed discordant species-level BLAST assignments that did not converge at the species- or clade-level on manual curation (e.g., *tef1* matching one species complex while *rpb2* matched a different one, with no clade-level convergence), thus failing the congruence criterion. Notably, JSRA09C1 and JSRA25x were recovered from the same sampling points as the isolates JSRA09 and JSRA25, respectively, but corresponded to morphologically distinct *Trichoderma* colonies rather than technical duplicates of the same culture; both were therefore screened independently and excluded under the above criteria (JSRA25x for a missing ITS read; JSRA09C1 for non-convergent multilocus species assignment despite complete three-marker data). All eleven excluded isolates were removed from downstream analyses to avoid uncertain identifications; the full per-isolate concordance analysis is presented in Supplementary Table S4a,b. Five isolates (JSRA09, JSRA16, JSRA22, JSRA28, JSRA30) met both criteria and were carried forward for phylogenetic and Cd tolerance characterization. We prioritized identification confidence over taxonomic breadth, following the recommendation of Cai and Druzhinina [12] that species-level assignments within *Trichoderma* require congruent multilocus evidence. The sequences generated for the five retained isolates were deposited in GenBank under the following accession numbers: ITS (PZ072289–PZ072293), *tef1* (PZ102226–PZ102230) and *rpb2* (PZ102231–PZ102235).

### 2.5. Phylogenetic Analysis

The nucleotide sequences from the three markers (ITS, *tef1*, *rpb2*) were edited and assembled using Sequencher v.5.4.6 (Gene Codes Corporation, Ann Arbor, MI, USA). Molecular identification followed the criteria established by Cai and Druzhinina [12] for the genus *Trichoderma*: generic assignment confirmed ≥76% similarity in ITS, and species-level identification based primarily on *tef1* and *rpb2*. Sequences were compared against the NCBI nucleotide database using BLASTn, and similarity percentages were recorded for each marker.

Because ITS shows limited taxonomic resolution at the species level within *Trichoderma* [12], a multilocus phylogenetic approach based on independent trees per marker was adopted, instead of a concatenated analysis. This strategy allowed for the congruence of the phylogenetic signal between loci to be assessed and avoided the dilution of phylogenetic resolution associated with concatenating markers with disparate evolutionary rates. Species-level identification was based primarily on the *tef1* and *rpb2* trees, while the ITS tree was included as a complementary reference at the genus level.

For each marker, the sequences generated in this study were aligned with reference sequences of *Trichoderma*-type strains retrieved from GenBank using MUSCLE implemented in MEGA v12.0.11 [18]. Alignments were inspected manually and ambiguous ends were trimmed prior to phylogenetic analysis. Reference sequences were prioritized from ex-type, ex-holotype or ex-epitype strains where available; for several species, the voucher associated with the curated ITS Reference Sequence (NCBI Reference Sequence, NR_ prefix) differs nominally from the voucher of the *tef1* and *rpb2* sequences (typically GJS-coded depositions by Samuels and collaborators), although both correspond to the same biological strain (e.g., *T. koningiopsis* CBS 119075 = GJS 93-20, both cultures from holotype BPI 802571B). For three species (*T. ovalisporum*, *T. pyramidale*, *T. viridescens*), the ITS sequence of the ex-type strain was not available in GenBank and the ITS of a different validated isolate was used, in agreement with the species-validation protocol of Cai and Druzhinina [12], which prioritizes *tef1* and *rpb2* for species-level identification. Complete accession numbers and voucher equivalences per marker are detailed in Supplementary Table S3.

Phylogenetic trees were inferred by Maximum Likelihood in MEGA 12, using a partial-deletion option with a 95% site-coverage threshold, which yielded 462 informative positions for ITS, 170 for *tef1* and 741 for *rpb2*. The Kimura 2-parameter (K2P) model with gamma-distributed rates among sites (5 discrete categories) was applied to the ITS dataset, and the General Time Reversible model with gamma-distributed rates (GTR+G, 5 discrete categories) was applied to the *tef1* and *rpb2* datasets. Initial trees for the heuristic search were obtained automatically by Neighbour-Joining and Maximum Parsimony (10 MP-tree searches), and the heuristic search was refined by Nearest-Neighbour-Interchange (NNI). Best-tree log-likelihoods were −1054.11 (ITS), −1399.64 (*tef1*) and −3403.91 (*rpb2*); estimated gamma shape parameters were α = 1.0269 (ITS), α = 0.8950 (*tef1*) and α = 0.2434 (*rpb2*). Node support was assessed with 1000 standard bootstrap replicates [19]. Trees were visualized in MEGA 12 and edited in iTOL v.6 [20]. Nodes with bootstrap support below 50% were considered unresolved and the corresponding phylogenetic relationships were interpreted with caution [21].

### 2.6. In Vitro Cadmium Tolerance Assays

Cadmium chloride (CdCl_2_) solutions were prepared at concentrations of 25, 50, 100, 150 and 200 ppm from a 1000 ppm stock solution. The solutions were adjusted to pH 7.0 ± 0.2 and sterilized by autoclaving (121 °C, 25 min). The sterile solutions were incorporated into molten PDA medium at 45–50 °C and poured into sterile Petri plates (90 mm diameter) immediately before solidification.

Inoculation was performed by central puncture with a sterile inoculation loop, depositing a small mycelial mass taken directly from fresh pure cultures (4–5 days on PDA, 28 °C) at the centre of each plate with Cd-supplemented medium. A control treatment without Cd (0 ppm) was included. Each strain × concentration combination had three independent replicates. Plates were incubated at 28 °C under a 12 h photoperiod for 24 days. Radial growth was measured with a digital calliper (nominal resolution ± 0.01 mm) in two perpendicular directions on days 1, 3, 7, 15 and 24 post-inoculation; measurements were recorded rounded to the nearest whole millimetre for consistency with the visual observation of the irregular colonial borders [22]. The complete raw dataset, comprising all individual replicate measurements per strain, Cd concentration and sampling day, is provided in Supplementary Table S2.

### 2.7. Tolerance Parameters

#### 2.7.1. Radial Growth Rate (RGR)

RGR (mm day^−1^) was calculated as RGR = D_final_/t_final_, where D_final_ is the diameter at day 24 and t_final_ = 24 days. D_0_ ≈ 0 mm was assumed because puncture inoculation deposited a punctual mycelial mass, not a preformed disc of standardized diameter [22].

#### 2.7.2. Growth Inhibition Percentage (GIP)

GIP was calculated according to Edgington et al. [23]: GIP (%) = [(D_c_ − D_t_)/D_c_] × 100, where D_c_ is the control diameter (0 ppm Cd) and D_t_ is the diameter of the Cd treatment at day 24. Positive values indicate growth inhibition relative to the control; negative values indicate marginal stimulation at low doses, attributable to biological variability within the measurement resolution (1 mm).

#### 2.7.3. Tolerance Index (TI)

TI (%) was calculated as TI = (D_t_/D_c_) × 100 = 100 − GIP [24].

#### 2.7.4. Dose–Response Model Selection and IC50 Estimation

Unlike the conventional approach that applies a single parametric model (e.g., four-parameter log-logistic, LL.4) to all data sets, the present study adopted a model-selection approach based on information theory [12]. This approach avoids the biases associated with imposing a single parametric structure on dose–response curves with heterogeneous shapes between strains [25,26].

For each strain, five families of models were fitted to the radial growth (day 24) as a function of Cd concentration: (i) four-parameter log-logistic (LL.4), (ii) three-parameter log-logistic (LL.3), (iii) four-parameter Weibull type 1 (W1.4), (iv) four-parameter Weibull type 2 (W2.4), and (v) simple linear regression. The non-linear fits (LL.4, LL.3, W1.4, W2.4) were performed exclusively on the Cd treatments (25–200 ppm; *n* = 5 dose levels), excluding the zero dose. The control (0 ppm) was used as a reference to calculate relative growth inhibition (GIP; Section 2.7.2) and as the implicit upper asymptotic limit in the four-parameter models, not as a data point on a logarithmic dose scale [27]. The linear model was fitted with the same structure to ensure comparability of AICs between families.

The best model per strain was selected using AIC, choosing the model with lowest AIC as representative of each strain’s dose–response relationship (Supplementary Table S1). When several models showed equivalent AICs (ΔAIC < 2) [28], selection privileged the most parsimonious model. Non-linear fits were performed with the drc v3.0-1 package in R [27].

The median inhibitory concentration (IC_50_) was estimated, with its 95% confidence intervals calculated by the delta method [27]. Following the recommendations of Ritz et al. [27] and Keshtkar et al. [25], IC_50_ is reported in two complementary formulations: (i) relative IC_50_, corresponding to the dose at 50% of the c–d asymptotic range of the model (parameter *e* by default in drc, type = “relative”); and (ii) absolute IC_50_, corresponding to the dose at which growth is reduced to 50% of the control value (type = “absolute” in drc). IC_50_ values were reported only when they fell within the experimental range tested (0–200 ppm); when the model predicted IC_50_ > 200 ppm, this is reported explicitly as out-of-range, and the tolerance interpretation is based on the observed GIP [25].

#### 2.7.5. Strain Classification According to Tolerance

Because IC_50_ is not interpretable when the response is nearly flat (GIP < 10% at the maximum tested dose), tolerance classification was performed using the GIP observed at 200 ppm as the primary empirical criterion: highly tolerant (GIP < 10%); moderately tolerant (10% ≤ GIP < 20%); tolerant (20% ≤ GIP < 35%); and sensitive (GIP ≥ 35%). This classification, based on observed data rather than extrapolated parameters, avoids the inconsistencies derived from estimating IC_50_ outside the experimental range [27].

### 2.8. Statistical Analysis

Radial growth data (mm) were summarized as mean ± standard error (SE) for each combination of strain, Cd concentration and incubation time, considering three independent replicates per treatment. In groups with zero variance between replicates, SE was 0.0.

Because multiple treatment groups showed zero variance among the three replicates (mainly at 25–50 ppm), formal parametric comparisons (ANOVA, *t*-tests) were not applicable due to a structural violation of the non-zero variance assumption. Inference was based on three complementary approaches: (i) AIC-based dose–response model selection as structural inference [12,27]; (ii) 95% confidence intervals for IC_50_ via the delta method (when this parameter fell within the experimental range); and (iii) Spearman’s correlation coefficient (ρ) between Cd concentration and radial growth at day 24, calculated on the means per strain across the five Cd treatments (25–200 ppm), with *p*-values obtained by exact permutation test given the small sample size (*n* = 5 dose levels; minimum achievable *p*-value for *n* = 5 is 0.0167), avoiding the asymptotic approximation that diverges artificially for perfect correlations. The control (0 ppm) was excluded from this correlation because it serves as the biological reference for GIP calculation rather than as a comparable point on the Cd dose scale; consequently, the reported correlations describe monotonicity strictly within the 25–200 ppm range, not across the full 0–200 ppm range.

Validation of the assumptions of the selected models was performed through residual diagnostic plots (QQ-plots and residuals vs. fitted values; Supplementary Figures S1 and S2). Temporal patterns of tolerance were analyzed by hierarchical clustering (Ward.D2 method, Euclidean distance) [29] represented in a heatmap (Supplementary Figure S3). All analyses were performed in R 4.5.2 [30] using the packages drc v3.0-1 [27], ggplot2 v4.0.2 [31], pheatmap v1.0.13 [32] and dplyr v1.2.0 [33]. The full annotated R script is provided as Supplementary Materials.

The sample size (*n* = 3 independent replicates per treatment) is standard for in vitro fungal tolerance screenings. A post hoc power analysis (two-tailed *t*-test, *n* = 3 per group, α = 0.05, Cohen’s d = 1.2) indicated an achieved power of approximately 0.21, well below the conventional 0.80 threshold. This sample size has limited power to detect moderate treatment differences via classical parametric tests; for this reason, inference in this study relies primarily on AIC-based dose–response modelling and on the Spearman correlation analysis described above, rather than on pairwise hypothesis testing. The limitations associated with this sample size are explicitly addressed in Section 4.5.

## 3. Results

### 3.1. Isolation and Distribution by Host Plant

Of the 36 rhizosphere samples collected across cocoa agroforestry plots in Bagua (Imaza and Copallín districts), 16 yielded *Trichoderma*-like isolates on selective PDA medium (Supplementary Table S4); the remaining 20 samples produced colonies of other soil fungi (mainly *Fusarium*, *Mucor*, *Alternaria* and *Aspergillus*) and were not retained for downstream analyses. Of these 16 *Trichoderma*-like isolates, five met the stringent multilocus identification criteria of Cai and Druzhinina [12] described in Section 2.4 (high-quality sequencing in the three markers and congruent multilocus assignments) and were retained for phylogenetic and Cd tolerance characterization (JSRA09, JSRA16, JSRA22, JSRA28, JSRA30). Four of the retained strains (JSRA16, JSRA22, JSRA28 and JSRA30) were isolated from the rhizosphere of *Inga* sp. (Fabaceae), and one strain (JSRA09) from the rhizosphere of *Quararibea* sp. (Malvaceae) (Table 1). It is noteworthy that the three rhizosphere samples collected from *Quararibea* sp. all yielded *Trichoderma*-like isolates, and one of them (JSRA09) passed the multilocus retention criteria; this high recovery rate, despite the limited number of *Quararibea* samples available (*n* = 3, reflecting the lower density of this species in the agroforestry plots; Section 2.2), suggests a consistent association that deserves further investigation. JSRA09 constitutes the first report of *Trichoderma* isolated from the rhizosphere of *Quararibea* sp. in Amazonian cocoa agroecosystems.

Identification based on a multilocus approach (ITS, *tef1*, *rpb2*) according to the criteria of Cai and Druzhinina [12], with independent Maximum Likelihood trees per marker. Sequences were deposited in GenBank.

### 3.2. Morphological Characterization

**Figure 2:**
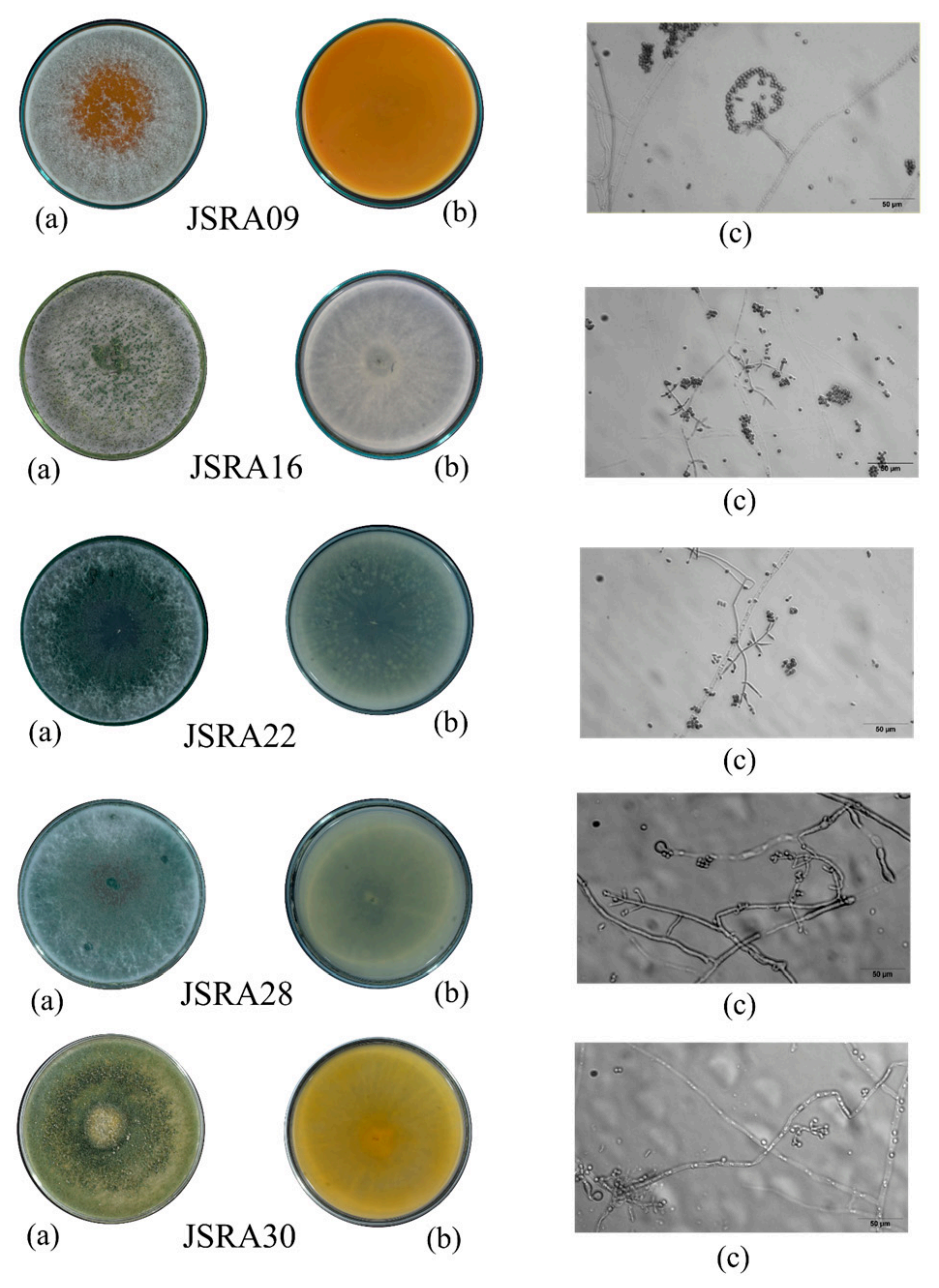
Macroscopic and microscopic morphology of the five *Trichoderma* strains isolated from rhizosphere soils of cocoa agroecosystems in Bagua, Amazonas, Peru. Rows, from top to bottom: JSRA09, JSRA16, JSRA22, JSRA28, JSRA30. Within each row: (**a**) colony front view; (**b**) colony reverse view; (**c**) microscopic morphology (400×) showing conidiophores and conidia. Scale bar = 50 µm (microscopy panels).

The five strains showed macroscopic and microscopic features consistent with the genus *Trichoderma*. At the macroscopic level, colonies exhibited rapid growth on PDA at 28 °C, with colour ranging from white at early stages to deep green, olive green and yellow-green according to the strain and incubation time. JSRA09 and JSRA28 reached diameters above 80 mm under control conditions at day 24, while JSRA22 showed slower growth (76 mm in the control). At the microscopic level, branched conidiophores with lageniform to ampulliform phialides (5.0–10.0 µm), globose to ellipsoidal conidia (2.5–4.5 × 2.0–3.5 µm) and intercalary chlamydospores in JSRA16 and JSRA30 were observed (Figure 2).

### 3.3. Molecular Identification and Multilocus Phylogenetic Analysis

**Figure 3:**
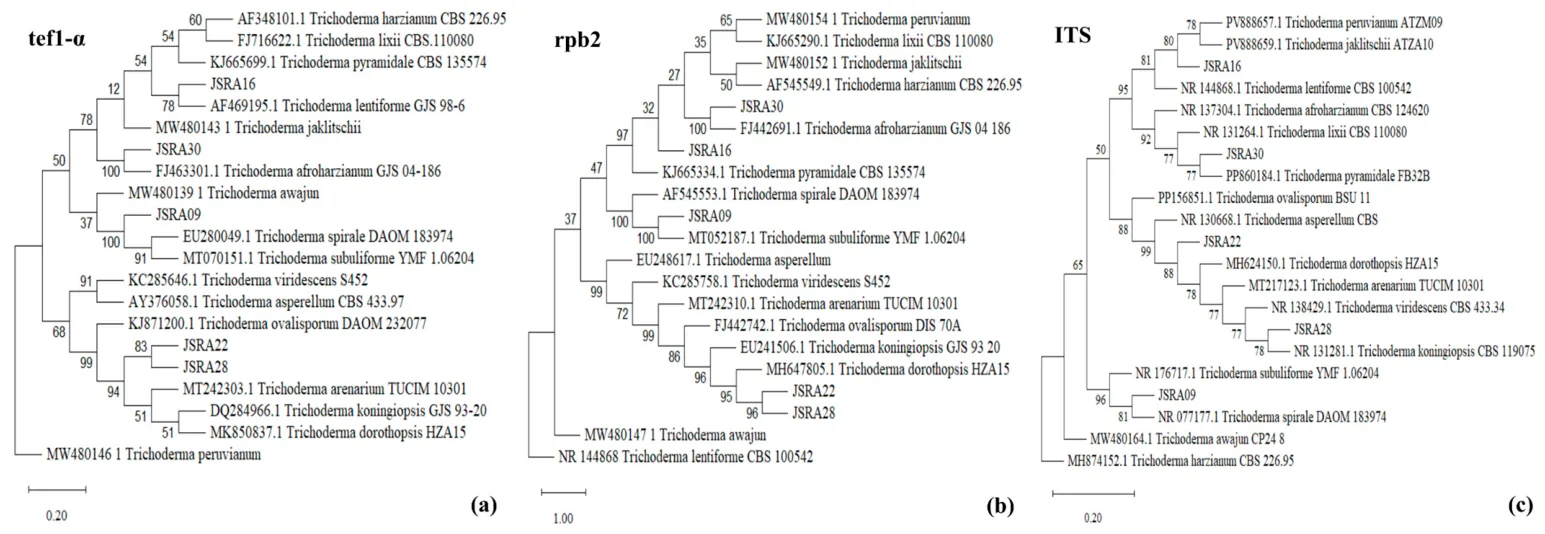
Maximum Likelihood phylogenetic trees per individual molecular marker: (**a**) *tef1*; (**b**) *rpb2*; (**c**) ITS. Phylogenetic positions of the five *Trichoderma* strains (JSRA09, JSRA16, JSRA22, JSRA28, JSRA30) are shown relative to reference sequences obtained from GenBank (Supplementary Table S3). Bootstrap support values (1000 replicates) ≥ 50% are shown above the nodes; nodes with bootstrap < 50% are considered unresolved. Evolutionary models: K2P+G for ITS (α = 1.0269; 462 positions); GTR+G for *tef1* (α = 0.8950; 170 positions) and *rpb2* (α = 0.2434; 741 positions).

Molecular identification through the multilocus approach (ITS, *tef1*, *rpb2*), following Cai and Druzhinina [12], allowed for taxonomic assignment of the five strains. The *tef1* and *rpb2* trees were inferred independently from non-overlapping alignments and yielded mutually consistent species-level placements for all focal strains, although fine-scale topologies and bootstrap supports differed between markers; the ITS tree (Figure 3c), retained as a complementary reference at the genus level, displayed a partially discordant topology that is consistent with the known low species-level resolution of ITS within *Trichoderma* [12].

JSRA09 was placed in a strongly supported clade with *T. spirale* and *T. subuliforme* in both *tef1* and *rpb2* (bootstrap = 100% in both markers). The ITS tree confirmed this placement (BS = 96% for the *T. spirale*/*T. subuliforme* + JSRA09 clade), reinforcing its assignment to a distinct lineage within this clade.

JSRA16 clustered with *T. lentiforme* in *tef1* (BS = 79%) within the *T. harzianum sensu lato* complex, and the ITS tree placed it in a clade including *T. lentiforme*, *T. peruvianum* and *T. jaklitschii* with moderate support (BS = 82%). In the *rpb2* tree, JSRA16 fell within a broader clade that includes *T. afroharzianum*, *T. jaklitschii*, *T. harzianum* and *T. lixii* (BS = 97%) without forming a direct sister relationship with *T. lentiforme*. Given this partial discordance, JSRA16 is reported as *Trichoderma* sp. within the *T. harzianum* complex, with affinity to *T. lentiforme*.

JSRA22 and JSRA28 formed a strongly supported monophyletic clade in both *tef1* (BS = 84%) and *rpb2* (BS = 97%). This pair was nested within a broader clade of the *T. koningii*/*T. koningiopsis* species complex *sensu lato*, comprising *T. dorothopsis*, *T. koningiopsis* and *T. arenarium* (BS = 94% in *tef1*; 99% in *rpb2*); the immediate sister relationship of the JSRA22 + JSRA28 clade with *T. arenarium* received weak support (BS = 18% in both *tef1* and *rpb2*). *T. peruvianum* MW480146 occupied a basal position in *tef1* (close to the outgroup) and was not phylogenetically adjacent to JSRA22 + JSRA28; therefore, the previously suggested affinity of these strains with *T. peruvianum* is not supported by the present analyses. The ITS tree did not recover JSRA22 and JSRA28 as a monophyletic clade (JSRA22 grouped with *T. dorothopsis*, BS = 88%; JSRA28 grouped with *T. koningiopsis*, BS = 78%), which is consistent with the limited species-level resolution of ITS within this complex [12]. Given the strong support for monophyly in the protein-coding markers but the weak resolution of the immediate sister relationship, JSRA22 and JSRA28 are reported as *Trichoderma* sp. of the *T. koningii*/*T. koningiopsis* complex, with the closest affinity to the *T. dorothopsis*/*T. arenarium* lineage.

JSRA30 clustered with *T. afroharzianum* GJS 04-186 with maximum support in both *tef1* and *rpb2* (BS = 100%). The ITS tree placed JSRA30 within a clade including *T. afroharzianum*, *T. lixii* and *T. pyramidale* (BS = 92%), with a sister relationship with *T. pyramidale* FB32B (BS = 77%) rather than with *T. afroharzianum*; this discordance is again attributable to the lower resolution of ITS at the species level. Following Cai and Druzhinina [12], the *tef1* and *rpb2* placements were prioritized and JSRA30 is identified as *T. afroharzianum*.

Overall, the results indicate a heterogeneous taxonomic composition: three strains (JSRA09, JSRA16, JSRA30) are assigned to cosmopolitan or widely distributed lineages, while JSRA22 and JSRA28 form a strongly supported monophyletic pair embedded within the *T. koningii*/*T. koningiopsis* complex. Voucher correspondences and complete accession numbers per marker are reported in Supplementary Table S3.

### 3.4. Radial Growth Response to Cadmium

The radial growth of the five strains was monitored for 24 days on PDA supplemented with Cd (25–200 ppm) (Supplementary Figure S5). All strains achieved stable growth on control plates (0 ppm) by day 24, with final diameters between 76.00 mm (JSRA22) and 82.00 mm (JSRA30) (Table 2). JSRA22 showed slower initial growth than the other strains during the first days, reaching its asymptote later; this dynamic was also reflected in greater variability between control replicates at day 24 (SE = 6.56 mm vs. ≤2.60 mm for the other strains) and is discussed in detail in Section 4.5.

The Cd response was strain-specific. JSRA09 showed the highest growth stability, maintaining diameters between 78 and 84 mm at day 24 across all concentrations, with a maximum GIP of only 1.67% at 200 ppm. JSRA16 exhibited similar tolerance, with maximum GIP of 5.86% at 200 ppm and diameters between 75 and 79 mm. JSRA28 and JSRA30 showed moderate inhibition only at concentrations ≥150 ppm, with maximum GIP values of 13.52% and 13.82% at 200 ppm, respectively. JSRA22 was the strain with the highest radial inhibition: GIP = 33.33% at 200 ppm and diameters reduced to 50–63 mm in the 100–200 ppm range (Table 2).

At low doses (25–50 ppm), JSRA09, JSRA28 and JSRA30 showed slightly negative GIP values (between −5.4% and −1.2%), corresponding to diameters 1–4 mm above the control. These oscillations were interpreted as biological variability within the measurement resolution (1 mm) rather than a structured hormetic response, given that (i) they occur within the rounding margin applied to the measurements and (ii) they do not maintain a monotonic pattern across the dose range. Brain–Cousens hormesis models were tested and rejected (*p* > 0.05) for all strains.

### 3.5. Selected Dose–Response Models and Estimated IC50 Values

For each strain, five families of models were fitted to the day-24 radial growth as a function of Cd concentration (excluding the 0 ppm control; Section 2.7.4). AIC-based selection revealed that no parametric family adequately describes all five strains (Supplementary Table S1), supporting the multi-model approach (Table 3). As a robustness check for the small number of dose levels (*n* = 5), the second-order corrected criterion (AICc) was also computed and is reported alongside AIC in Supplementary Table S1; AICc rankings were concordant with AIC for all strains, with the exception of a negligible reversal for JSRA22 (ΔAICc < 0.01 between the linear and LL.4 models), which does not affect the practical interpretation given the AIC-based model equivalence already noted [28]. For JSRA09 and JSRA16, the linear model showed the lowest AIC (64.07 and 65.19, respectively), consistent with the nearly flat responses within the tested range. For JSRA22, JSRA28 and JSRA30, the four-parameter log-logistic model (LL.4) showed the lowest AIC (71.60, 41.83 and 30.01, respectively); for JSRA22 and JSRA28, the LL.4, W1.4 and W2.4 families were statistically equivalent (ΔAIC ≤ 0.01), and LL.4 was retained as the lowest-AIC family and for consistency across the three non-linear strains.

For JSRA22, JSRA28 and JSRA30, the AIC ties between three or more model families (ΔAIC ≤ 0.5) imply that the data do not allow structural discrimination between them. The reported IC_50_ values represent the best estimate of the model selected by parsimony, but equivalent estimates would be obtained with the competing models. This structural indeterminacy reinforces the argument that no single parametric model is universally appropriate to describe the dose–Cd response in heterogeneous strains [12].

**Figure 4:**
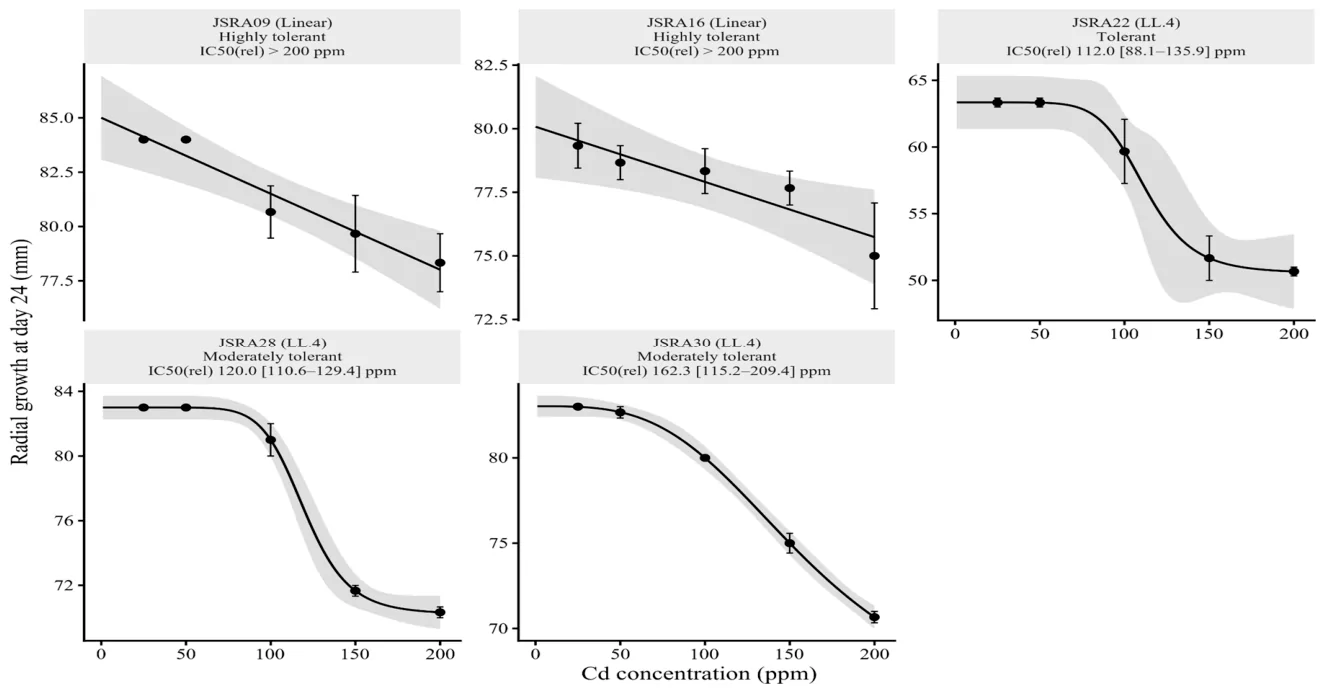
Dose–response curves and IC_50_ estimates for the five *Trichoderma* strains using the model selected by AIC for each strain (linear for JSRA09 and JSRA16; LL.4 for JSRA22, JSRA28 and JSRA30), based on day-24 radial growth. Points represent observed means ± SE; shaded areas represent 95% confidence intervals calculated by the delta method. Relative IC_50_ values (ppm) and tolerance classification are indicated above each panel. For JSRA09 and JSRA16, the linear model produces nearly flat responses with IC_50_ > 200 ppm (extrapolated outside the experimental range; reported as “IC_50_ > 200 ppm” in the panels).

Relative IC_50_ fell within the tested experimental range for three of the five strains: JSRA22 (LL.4): 112.0 [88.1–135.9] ppm; JSRA28 (LL.4): 120.0 [110.6–129.4] ppm; JSRA30 (LL.4): 162.3 [115.2–209.4] ppm. Under the absolute formulation, IC_50_ exceeded 200 ppm in all five strains, since none reached 50% inhibition of control growth within the experimental range (maximum GIP: 33.33% in JSRA22). For JSRA09 and JSRA16, both formulations agreed in IC_50_ > 200 ppm (Table 3). The fitted dose–response curves and the corresponding relative IC_50_ estimates for each strain are shown in Figure 4.

### 3.6. Monotonic Association Between Cd and Radial Growth

The Spearman correlation between Cd concentration and radial growth at day 24 was strong and significant for all strains, indicating a monotonic decreasing trend of growth with increasing Cd dose (Table 4). The five strains showed ρ coefficients between −0.975 and −1.000, with *p*-values obtained by exact permutation test (*n* = 5; for *n* = 5, the minimum achievable two-tailed *p*-value is 0.0167, corresponding to ρ = ±1.000). *p*-values ranged between 0.0167 and 0.0333, confirming that, despite the structural differences between the selected parametric models (Table 3), the direction of the Cd effect on growth is consistent.

Reported *p*-values correspond to two-tailed exact permutation tests. For *n* = 5, the minimum possible *p*-value is 1/60 = 0.0167, so statistical significance is bounded below by the sample size.

### 3.7. Diagnostics of Selected Models

Diagnostic plots (QQ-plots and residuals vs. fitted values; Supplementary Figures S1 and S2) showed reasonable fits for JSRA09, JSRA16, JSRA28 and JSRA30, with residuals close to the theoretical normal line and symmetric dispersion around zero. For JSRA22, moderate deviations were observed in the QQ-plot tails, attributable to the higher variability between replicates at 100–150 ppm (SE = 1.67–2.40 mm) and to the presence of an extreme residual point near 100 ppm. JSRA28 and JSRA30 showed near-zero variance in several treatments (residuals ≈ 0), which constitutes an artefact of the rounding to whole millimetres rather than a favourable statistical property; this aspect is addressed in Section 4.5 (Limitations of the Study).

### 3.8. Intraspecific Variability: JSRA22 vs. JSRA28

JSRA22 and JSRA28, both placed in a strongly supported monophyletic clade in *tef1* (BS = 84%) and *rpb2* (BS = 97%; Figure 3), showed markedly contrasting Cd responses (Supplementary Figure S4). At 200 ppm, the GIP of JSRA22 (33.33%) was 2.5 times higher than that of JSRA28 (13.52%), and the radial diameters at day 24 were 50.67 mm vs. 70.33 mm, respectively. This divergence is maintained across the tested range: at 100 ppm, JSRA22 already shows GIP = 21.49%, while JSRA28 maintains GIP ≤ 0.5%. The phylogenetic similarity between both strains and the observed functional divergence evidence intraspecific variability in Cd response, suggesting that tolerance is strain-specific and not necessarily predictable from close phylogenetic affiliation.

## 4. Discussion

### 4.1. First Report of Trichoderma in the Rhizosphere of Quararibea *sp.*

The isolation of strain JSRA09 from the rhizosphere of *Quararibea* sp. constitutes, to the best of our knowledge based on the reviewed literature, the first report of *Trichoderma* associated with this host species in Amazonian cocoa agroecosystems. Previous studies on *Quararibea cordata* have focused on the mineral composition of its fruits [6] and not on its rhizosphere microbiome. This result expands the range of rhizosphere hosts described for the genus in the region, complementing previous reports in *Inga* sp. and *T. cacao* [4,11]. The identification of JSRA09 within the *T. spirale*/*T. subuliforme* clade suggests that *Quararibea* sp. may harbour phylogenetically distinct lineages from those usually reported in cocoa (typically the *T. harzianum* complex) [9]. This finding justifies further investigations with broader sampling on *Quararibea* sp. in other Amazonian areas to evaluate the consistency of the association.

### 4.2. Taxonomic Composition and Phylogenetic Interpretation

The multilocus identification using independent trees per marker, instead of a concatenated analysis, allowed for assessment of the congruence of the phylogenetic signal between loci and refinement of the taxonomic assignments. This strategy is particularly relevant in *Trichoderma*, where ITS shows limited resolution at the species level [12]; concatenated analysis can dilute the signal of markers with greater discriminatory power (*tef1*, *rpb2*) when combined with markers of lower resolution. The agreement between *tef1* and *rpb2* at the level of focal species placements supports species-level identifications, while the partial discordance with the ITS topology is consistent with the documented limitations of ITS for fine-scale resolution in this genus.

The results show a heterogeneous taxonomic composition: three strains are assigned to cosmopolitan or widely distributed lineages (JSRA09 to the *T. spirale*/*T. subuliforme* clade; JSRA16 to the *T. harzianum sensu lato* complex with affinity to *T. lentiforme*; and JSRA30 to *T. afroharzianum*), while JSRA22 and JSRA28 form a strongly supported monophyletic pair embedded within the *T. koningii*/*T. koningiopsis* species complex *sensu lato*. The immediate sister relationship of the JSRA22 + JSRA28 clade with *T. arenarium* received weak bootstrap support (BS = 18% in both protein-coding markers), and the basal position of *T. peruvianum* in the *tef1* and *rpb2* trees indicates that these strains are not specifically related to *T. peruvianum*, contrary to what an ITS-only analysis might initially suggest. The initial hypothesis posited phylogenetic affinity with Peruvian endemic species; the present results show that this hypothesis is only partially supported, since the JSRA22 + JSRA28 clade is associated with the broader *koningii*/*koningiopsis* complex, which has a wide geographic distribution and is not endemic to Peru. Additional sampling and population-level analyses would be needed to clarify whether the JSRA22 + JSRA28 lineage represents a regionally restricted variant within this complex or a broader cosmopolitan lineage.

### 4.3. Cadmium Tolerance and Validity of the Multi-Model Approach

All five strains showed Cd tolerance within the tested range: none exceeded 35% radial growth inhibition at 200 ppm (maximum GIP: 33.33% in JSRA22). This tolerance range is comparable to that reported for *Trichoderma* strains in previous studies on heavy-metal-contaminated soils [8,10] and suggests that Amazonian cocoa rhizospheres select Cd-tolerant microbiota in vitro. The classification based on observed GIP at 200 ppm distributed the strains into three of the four predefined categories: highly tolerant (JSRA09 and JSRA16, GIP < 10%), moderately tolerant (JSRA28 and JSRA30, 10% ≤ GIP < 20%) and tolerant (JSRA22, 20% ≤ GIP < 35%); no strain fell into the sensitive category (GIP ≥ 35%) according to the primary classification, although JSRA22 lies at the boundary between the Tolerant and Sensitive categories and its classification is sensitive to the control aggregation method (see Section 4.5).

AIC-based model selection revealed that no parametric family adequately describes the dose–effect responses of the five strains (Table 3). This structural heterogeneity supports the methodological decision to adopt a multi-model approach [12,25,27] instead of applying a single parametric model—typically LL.4—to all data, as is common practice in fungal tolerance studies. The linear models selected for JSRA09 and JSRA16 reflect nearly flat responses (diameter changes ≤ 6 mm in the 0–200 ppm range), where an extrapolated IC_50_ outside the tested range lacks direct biological interpretation. In contrast, the non-linear models (LL.4 for JSRA22, JSRA28 and JSRA30) allowed for relative IC_50_ to be estimated within the experimental range.

The divergence between relative IC_50_ and absolute IC_50_ for JSRA22, JSRA28 and JSRA30 (Table 3) is expected: under the relative formulation, IC_50_ is interpreted as the midpoint of the asymptotic range estimated by the model, while under the absolute formulation it requires the response to fall to 50% of the control, which does not occur within the tested range in any strain. This divergence illustrates the importance of specifying the type of IC_50_ reported in tolerance studies [27]. For JSRA22, JSRA28 and JSRA30, the AICs of several model families were equivalent (ΔAIC < 2). According to the criterion of Burnham and Anderson [28], this indicates that multiple models are substantially equivalent in describing the data, and the selection of a single model represents a parsimony decision rather than a strict statistical discrimination.

### 4.4. Intraspecific Variability Between JSRA22 and JSRA28

The functional divergence observed between JSRA22 and JSRA28, two phylogenetically close strains forming a strongly supported monophyletic clade in *tef1* (BS = 84%) and *rpb2* (BS = 97%), constitutes a relevant finding for the selection of candidates in future bioremediation screening programmes. JSRA22 showed twice the radial inhibition of JSRA28 at 200 ppm (33.33% vs. 13.52%), despite their genetic similarity in the three molecular markers evaluated. This pattern is consistent with previous observations in *Trichoderma* and other filamentous fungi [8], where heavy-metal tolerance is a strain-specific property influenced by factors not fully captured by universal phylogenetic markers (e.g., variation in metal transporters, extracellular chelating capacity, differential expression of genes associated with oxidative stress). This intraspecific variability reinforces the importance of individual functional evaluation per strain prior to incorporation into microbial consortia, as affiliation with the same nominal species does not guarantee equivalent functional properties.

### 4.5. Limitations of the Study

The present study has seven limitations that deserve explicit consideration and that orient the interpretive scope of the results.

First, there was unbalanced sampling between host species and the final number of characterized strains. The 36 rhizosphere samples were unevenly distributed between host species (31 *Inga* sp. vs. 3 *Quararibea* sp.), reflecting the natural composition of the agroforestry plots (where *Inga* sp. is the dominant shade tree species and *Quararibea* sp. occurs at lower density) rather than an experimental design choice. Although all three *Quararibea* samples yielded *Trichoderma*-like isolates (and one fulfilled the multilocus retention criteria, supporting the first report of the association), the small sample size for this host limits the inference about the breadth and consistency of the *Trichoderma*–*Quararibea* association. In addition, of the 16 *Trichoderma*-like isolates ultimately recovered, only five met the stringent multilocus retention criteria (ITS, *tef1*, *rpb2*) of Cai and Druzhinina [12]: eight isolates were excluded due to sequencing quality below the Q ≥ 50% threshold for one or more markers, and three isolates produced high-quality sequences in all three markers but showed discordant species-level BLAST assignments that did not converge at the species- or clade-level; two of these eleven (JSRA09C1 and JSRA25x) originated from the same sampling points as the retained isolates JSRA09 and JSRA25 but corresponded to morphologically distinct colonies, not technical duplicates (Supplementary Table S4a,b). We prioritized identification confidence over taxonomic breadth, as recommended by Cai and Druzhinina [12] for species-level assignments within *Trichoderma*. While this strategy strengthens the taxonomic robustness of the reported placements, it constrains inference about the full diversity of *Trichoderma* in the studied rhizospheres; future studies should consider a broader sampling design targeted at *Quararibea* sp. and complementary phylogenetic markers to resolve the species complexes encountered here.

Second, results were rounded to whole millimetres with zero variance between replicates. Colonial diameter measurements were performed with a digital calliper and recorded as rounded to the whole millimetre for consistency with the visual observation of irregular colonial borders. This rounding produces zero variance between replicates in some treatments (Table 2), which constitutes a structural artefact rather than a favourable statistical property. The direct consequence is the invalidation of the assumptions of parametric tests such as ANOVA and the impossibility of applying conventional *t*-tests, which forces the inference to be based on model selection, confidence intervals and non-parametric correlations. The slight negative GIP values at low doses (e.g., −5.4% in JSRA09 at 25 and 50 ppm) reflect this submillimetric variability rather than biological hormesis, as confirmed by the rejection of Brain–Cousens hormesis models (*p* > 0.05). The choice of the inferential approach responds to this structural limitation and does not imply a universal methodological preference.

Third, there was a small sample size (*n* = 3 replicates per treatment; *n* = 5 dose levels). The experimental design with three biological replicates per treatment limits the statistical power to detect moderate effects, and the *p*-values from the exact Spearman permutation test are bounded below at 0.0167 (*n* = 5). The sample size is standard for in vitro fungal heavy-metal tolerance screenings [8], but limits the generalization of the results and forces a cautious interpretation of differences between strains.

Fourth, there is extreme variance in the JSRA22 control and sensitivity of the classification. The three control replicates of JSRA22 at day 24 showed diameters of 63, 81 and 84 mm (SE = 6.56 mm), while its Cd treatments showed SE ≤ 2.40 mm. This heterogeneity reflects the slower initial growth characteristic of this strain, which stabilized in individual replicates with different dynamics. No methodological cause was identified that justified the exclusion of replicates (contamination, incubation failure, etc.), so the primary classification uses the mean of the three replicates. However, the sensitivity analysis shows that JSRA22 lies at the boundary between Tolerant and Sensitive categories (threshold GIP = 35%), and its final classification will depend on additional evaluations with extended replicates that allow for better characterization of its control growth pattern.

Fifth, central puncture inoculation with inoculation loop was used. Inoculation deposited a mycelial mass of non-standardized size (≈1 mm). This introduces initial variability between plates that is not controlled by a uniformly sized disc-inoculum. The central parameters of the study (GIP, TI, IC_50_) were calculated as ratios relative to the control of each strain, which is robust to the absolute size of the inoculum as long as the inoculum is homogeneous between plates of the same strain. However, absolute comparisons of growth rates between strains should be interpreted with caution.

Sixth, the range of Cd concentrations vs. real environmental levels and absence of mechanistic assays constitutes a limitation. The tested range (25–200 ppm of CdCl_2_ in PDA) covers Cd levels that greatly exceed the concentrations observed in cocoa soils of Bagua (1.02–3.54 mg kg^−1^) [1]. Although high doses are useful to discriminate differential tolerances between strains in vitro, the direct translation of IC_50_ to in situ effective levels requires confirmatory experiments under real edaphic conditions, considering factors such as soil pH, organic matter, Cd bioavailability and microbial competition. Furthermore, the present study evaluated only tolerance to Cd as measured by radial growth inhibition; mechanistic assays of Cd biosorption, bioaccumulation, siderophore production and plant-associated performance were not performed and are required to demonstrate bioremediation capacity per se. The present study is therefore conceived as a primary in vitro screening stage, and the results should guide future evaluations on naturally contaminated soils, plant-associated assays and, eventually, field trials.

Seventh, the Cd tolerance assays did not include a reference strain with known Cd tolerance (positive control) because the primary objective was the intrinsic screening of native strains without external bias. The Cd-free control (0 ppm) served as the internal reference for the calculation of the Growth Inhibition Percentage (GIP) and Tolerance Index (TI), and all experimental conditions (medium, temperature, incubation time) were kept strictly uniform across strains to ensure internal comparability.

### 4.6. Implications for Future Bioremediation Screening

The five strains show an in vitro Cd tolerance compatible with their inclusion in further screening programmes aimed at developing microbial consortia for bioremediation of Amazonian cocoa soils. However, the present results document Cd tolerance only in vitro on PDA supplemented with CdCl_2_ and should not be extrapolated as evidence of bioremediation capacity per se, since Cd removal, immobilization and plant-associated performance were not assessed in this study. Within this in vitro context, JSRA09 and JSRA16, with GIP < 10% at 200 ppm, are priority candidates due to their broad constitutive tolerance and to their representing distinct phylogenetic lineages (a clade close to *T. spirale*/*T. subuliforme* and the *T. harzianum* complex). JSRA30 (*T. afroharzianum*) offers moderate tolerance and belongs to a species already described as a bioremediation agent in other contexts [9]. JSRA22 and JSRA28, despite their lower relative tolerance, are interesting because they represent a strongly supported monophyletic pair within the *T. koningii*/*T. koningiopsis* complex and offer a natural framework to study the genetic basis of intraspecific variability in tolerance. The final selection of strains for microbial consortia in subsequent studies on naturally contaminated soils and plant-associated systems should also consider complementary functional properties (production of chelators, bioaccumulation capacity, antagonism against phytopathogens).

## 5. Conclusions

Five native *Trichoderma* strains representing four phylogenetic lineages were recovered from shade-tree rhizospheres in Amazonian cocoa agroecosystems. JSRA09, isolated from *Quararibea* sp., provides the first reported association of *Trichoderma* with this host in this setting.

Cadmium tolerance was strain-specific. At 200 ppm Cd, growth inhibition was 1.67% for JSRA09, 5.86% for JSRA16, 13.52% for JSRA28, 13.82% for JSRA30, and 33.33% for JSRA22. Absolute IC50 exceeded 200 ppm for all strains, although JSRA22 remains close to the predefined tolerant–sensitive boundary because of control variability.

These findings identify JSRA09 and JSRA16 as priority candidates for confirmatory screening. Their use in bioremediation cannot yet be inferred from radial-growth tolerance alone and requires measurements of Cd immobilization or uptake, followed by tests in naturally contaminated soils and plant-associated systems.

## Figures and Tables

**Table 1:** *Trichoderma* strains isolated from rhizosphere soil of cocoa plots in Bagua, Amazonas, Peru.

Strain	Host	District	Molecular Identification	GenBank ITS	GenBank *tef1*	GenBank *rpb2*
JSRA09	*Quararibea* sp.	Imaza	*Trichoderma* sp. (*T. spirale*/*T. subuliforme* clade)	PZ072289	PZ102226	PZ102231
JSRA16	*Inga* sp.	Copallín	*Trichoderma* sp. (*T. harzianum* complex; affinity to *T. lentiforme*)	PZ072290	PZ102227	PZ102232
JSRA22	*Inga* sp.	Copallín	*Trichoderma* sp. (*T. koningii*/*T. koningiopsis* complex; close to *T. dorothopsis*/*T. arenarium*)	PZ072291	PZ102228	PZ102233
JSRA28	*Inga* sp.	Copallín	*Trichoderma* sp. (*T. koningii*/*T. koningiopsis* complex; close to *T. dorothopsis*/*T. arenarium*)	PZ072292	PZ102229	PZ102234
JSRA30	*Inga* sp.	Copallín	*T. afroharzianum*	PZ072293	PZ102230	PZ102235

**Table 2:** Cadmium tolerance parameters of the five *Trichoderma* strains at day 24. Radial growth expressed as mean ± SE (mm); GIP = growth inhibition percentage; TI = tolerance index.

Strain	Cd (ppm)	Diameter (mm) ± SE	GIP (%)	TI (%)
JSRA09	0	79.67 ± 2.60	-	-
JSRA09	25	84.00 ± 0.00	−5.44	105.44
JSRA09	50	84.00 ± 0.00	−5.44	105.44
JSRA09	100	80.67 ± 1.20	−1.26	101.26
JSRA09	150	79.67 ± 1.76	0.00	100.00
JSRA09	200	78.33 ± 1.33	1.67	98.33
JSRA16	0	79.67 ± 1.20	-	-
JSRA16	25	79.33 ± 0.88	0.42	99.58
JSRA16	50	78.67 ± 0.67	1.26	98.75
JSRA16	100	78.33 ± 0.88	1.67	98.33
JSRA16	150	77.67 ± 0.67	2.51	97.49
JSRA16	200	75.00 ± 2.08	5.86	94.14
JSRA22	0	76.00 ± 6.56	-	-
JSRA22	25	63.33 ± 0.33	16.67	83.33
JSRA22	50	63.33 ± 0.33	16.67	83.33
JSRA22	100	59.67 ± 2.40	21.49	78.51
JSRA22	150	51.67 ± 1.67	32.02	67.98
JSRA22	200	50.67 ± 0.33	33.33	66.67
JSRA28	0	81.33 ± 1.67	-	-
JSRA28	25	83.00 ± 0.00	−2.05	102.05
JSRA28	50	83.00 ± 0.00	−2.05	102.05
JSRA28	100	81.00 ± 1.00	0.41	99.59
JSRA28	150	71.67 ± 0.33	11.89	88.12
JSRA28	200	70.33 ± 0.33	13.52	86.48
JSRA30	0	82.00 ± 1.53	-	-
JSRA30	25	83.00 ± 0.00	−1.22	101.22
JSRA30	50	82.67 ± 0.33	−0.81	100.81
JSRA30	100	80.00 ± 0.00	2.44	97.56
JSRA30	150	75.00 ± 0.58	8.54	91.46
JSRA30	200	70.67 ± 0.33	13.82	86.18

**Table 3:** Dose–response model selected by AIC, relative IC_50_, absolute IC_50_, GIP observed at 200 ppm, and tolerance classification.

Strain	Model	AIC	Relative IC_50_ (ppm)	Absolute IC_50_ (ppm)	GIP@200 (%)	Classification
JSRA09	Linear	64.07	>200	>200	1.67	Highly tolerant
JSRA16	Linear	65.19	>200	>200	5.86	Highly tolerant
JSRA22	LL.4	71.60	112.0 [88.1–135.9]	>200	33.33 ^a^	Tolerant ^a^
JSRA28	LL.4	41.83	120.0 [110.6–129.4]	>200	13.52	Moderately tolerant
JSRA30	LL.4	30.01	162.3 [115.2–209.4]	>200	13.82	Moderately tolerant

Relative IC_50_ = dose at 50% of the c–d asymptotic range of the model [27]. Absolute IC_50_ = dose at which growth is reduced to 50% of the control [25]. 95% confidence intervals calculated by the delta method. Tolerance classification is based on observed GIP at 200 ppm (Section 2.7.5), not on IC_50_. ^a^ Sensitivity Analysis for JSRA22: The three control replicates at day 24 showed high variability (63, 81 and 84 mm; SE = 6.56 mm), attributed to the slow initial growth characteristic of this strain that stabilized in individual replicates with different dynamics (Section 4.5). Using the mean of the three replicates (76.0 mm), GIP@200 = 33.33% and the classification is Tolerant. If the control median (81.0 mm) were used, GIP@200 = 37.45% and the classification would shift to Sensitive (GIP ≥ 35%); excluding the lowest-growth control replicate (63 mm), GIP@200 = 38.59% (also Sensitive). The primary classification reported is based on the mean of the three observed replicates.

**Table 4:** Spearman correlation coefficients between Cd concentration and radial growth at day 24, calculated on the means per strain (*n* = 5 dose levels), with *p*-values obtained by exact permutation test.

Strain	Spearman ρ	*p*-Value (Exact)	*n*	Significance	Magnitude
JSRA09	−0.975	0.0333	5	Yes (*p* < 0.05)	Strong
JSRA16	−1.000	0.0167	5	Yes (*p* < 0.05)	Strong
JSRA22	−0.975	0.0333	5	Yes (*p* < 0.05)	Strong
JSRA28	−0.975	0.0333	5	Yes (*p* < 0.05)	Strong
JSRA30	−1.000	0.0167	5	Yes (*p* < 0.05)	Strong

## Data Availability

The nucleotide sequences generated in this study were deposited in GenBank under accession numbers PZ072289–PZ072293 (ITS), PZ102226–PZ102230 (*tef1*) and PZ102231–PZ102235 (*rpb2*). All raw radial growth data, tolerance parameters (GIP, TI) and IC_50_ estimates are included in the manuscript and Supplementary Materials. The annotated R script used for dose–response model fitting and IC_50_ estimation is provided as Supplementary Materials. Additional data supporting the findings of this study are available from the corresponding author upon reasonable request.

## Supplementary Materials

The following supporting information can be downloaded at: https://www.mdpi.com/article/10.3390/jof12090644/s1. Figure S1: QQ-plots of residuals for the dose–response models selected by AIC; Figure S2: Plots of residuals vs. fitted values for the selected models; Figure S3: Heatmap with hierarchical clustering (Ward.D2 method, Euclidean distance) of radial growth of the five strains across days 1, 3, 7, 15 and 24 under the six Cd concentrations; Figure S4: Comparison of the Cd response between the two strains forming the strongly supported monophyletic clade nested within the *T. koningii*/*T. koningiopsis* complex (JSRA22 and JSRA28); Figure S5: Radial growth curves (mm) over 24 days for the five *Trichoderma* strains under 0–200 ppm Cd; Table S1: AIC comparison for the five dose–response models evaluated (LL.4, LL.3, W1.4, W2.4, Linear) per strain; Table S2: Raw radial growth data (mm) per strain, concentration, day and replicate; Table S3: GenBank reference sequences used in the phylogenetic analyses for each marker (ITS, tef1, rpb2), including voucher equivalences across markers; Table S4a: Initial 16 *Trichoderma*-like isolates recovered from the 36 rhizosphere samples (sampling origin, GPS coordinates, altitude, multilocus sequencing outcome, and inclusion/exclusion reason per isolate); Table S4b: Multilocus concordance analysis of the 11 excluded isolates following the species-validation protocol of Cai and Druzhinina (2021) [12], with *tef1* and *rpb2* best BLAST hits and per-marker concordance verdicts.
